# Protective role of down-regulated microRNA-31 on intestinal barrier dysfunction through inhibition of NF-κB/HIF-1α pathway by binding to HMOX1 in rats with sepsis

**DOI:** 10.1186/s10020-018-0053-2

**Published:** 2018-10-19

**Authors:** Cheng-Ye Zhan, Di Chen, Jin-Long Luo, Ying-Hua Shi, You-Ping Zhang

**Affiliations:** Intensive Care Unit, Tongji Hospital Affiliated to Tongji Medical College of Huazhong University of Science and Technology, No. 1095, Jiefang Road, Qiaokou District, Wuhan, 430030 Hubei Province People’s Republic of China

**Keywords:** microRNA-31, Sepsis, HMOX1, NF-κB/HIF-1α pathway, Intestinal barrier dysfunction

## Abstract

**Background:**

Intestinal barrier dysfunction is a significant clinical problem, commonly developing in a variety of acute or chronic pathological conditions. Herein, we evaluate the effect of microRNA-31 (miR-31) on intestinal barrier dysfunction through NF-κB/HIF-1α pathway by targeting HMOX1 in rats with sepsis.

**Methods:**

Male Sprague-Dawley rats were collected and divided into the sham group, and the cecum ligation and perforation group which was subdivided after CACO-2 cell transfection of different mimic, inhibitor, or siRNA. Levels of serum D-lactic acid, diamine oxidase and fluorescence isothiocyanate dextran, FITC-DX concentration, and bacterial translocation were detected. Superoxidedismutase (SOD) activity and malondialdehyde (MDA) content were evaluated using the colorimetric method and an automatic microplate reader, respectively. Additionally, the levels of tumor necrosis factor, interleukin (IL)-6, and IL-10 were tested using enzyme-linked immunosorbent assay. The expression of miR-31, HMOX1, NF-κB, HIF-1α, IκB, ZO-1 and Occludin were assessed by reverse transcription quantitative polymerase chain reaction and Western blot analysis.

**Results:**

Inhibition of miR-31 decreased intestinal mucosal permeability and intestinal barrier function. The increased levels of miR-31 could cause oxidative damage and affect the expression of inflammatory factors in intestinal tissue of rats. HMOX1 was confirmed as a target gene of miR-31. MiR-31 affected intestinal mucosal permeability and intestinal barrier function, as well as oxidative damage and inflammation level by regulating HMOX1. Down-regulation of miR-31 inhibited NF-κB/HIF-1α pathway related genes by regulating HMOX1 expression. Furthermore, inhibition of miR-31 increased survival rates of rats.

**Conclusion:**

Overall, the current study found that inhibition of miR-31 protects against intestinal barrier dysfunction through suppression of the NF-κB/HIF-1α pathway by targeting HMOX1 in rats with sepsis.

## Background

Sepsis, a life-threatening clinical syndrome, is characterized by the presence of both infection and a systemic inflammatory response (Tiruvoipati et al. 2010). The common clinical manifestations of sepsis are correlated with systemic inflammatory response syndrome and organ dysfunction, including hemodynamic instability, hypoxemia, and intestinal barrier dysfunction (Melvan et al. 2010). In addition, the intestine contains endogenous and exogenous microorganisms which have been reported to be the potential pathogens of sepsis, and it can also be susceptible to ischaemia-reperfusion injuries because of sepsis (Jiang et al. 2013). Moreover, intestinal barrier dysfunction can result in secondary bacterial translocation and various clinical syndromes of multiple organ dysfunctions in sepsis (Fredenburgh et al. 2011). Previous studies have demonstrated that anti-inflammatory genes targeting intestinal barrier dysfunction and relevant pathogenic factors can decrease bacterial translocation and unfavourable inflammatory responses, which thereby can increase survival rate in sepsis (Jiang et al. 2013). Importantly, microRNA (miRNA) dysregulation was reported to be correlated with clinical manifestations and inflammation, which thereby could serve as a potential therapeutic target against sepsis (Zhou et al. 2015).

MiRNAs are small noncoding RNAs, post-transcriptionally inhibiting target gene expression by base paring to their 3’untranslated region (3’UTR) (Bartel 2009). They are involved in gene regulatory networks managing almost all cellular functions, and are essential in some diseases like inflammation as well as cancer (Montano 2011; O'Connell et al. 2012; Schetter et al. 2010). Recently, miR-31 was confirmed to be a regulator of hypoxia-inducible factor (HIF)-1α and nuclear factor-kappa B (NF-κB) which was closely correlated key transcription factor families (Creighton et al. 2010; Valastyan et al. 2009). MiR-31 stimulates the HIF pathway by targeting factor-inhibiting HIF-1α (FIH) in human head and neck carcinoma (Liu et al. 2010), and inhibits the noncanonical NF-κB pathway in adult T cell leukemia by suppressing NF-κB–inducing kinase (NIK) (Yamagishi et al. 2012). Heme oxygenase (HMOX) 1 is essential in the defense of the body against oxidant-induced injury during inflammatory processes, in which elevated plasma concentrations of HMOX1 have been observed in septic patients and experimental models of sepsis syndrome (Takaki et al. 2010). As a cytoprotective enzyme, HMOX1 has anti-inflammatory, antioxidant, antiapoptotic, and antiproliferative effects (Hou et al. 2010). HMOX1 can be induced by oxidative stress-promoting agents such as heme, hyperoxia, hypoxia, heat shock, endotoxins, hydrogen peroxide, heavy metals, and nitric oxide (Vazquez-Armenta et al., 2013). A recent study has shown that lower expression of miR-31 in CD4+ T Cells contributed to immunosuppression in human sepsis by promoting TH2 skewing (van der Heide et al. 2016). From all above, it can be hypothesized that the miR-31, HMOX1, and the HIF-1α/NF-κB pathway exert certain effects on sepsis. Thereby, the current study aims to investigate the effect of miR-31 on intestinal barrier dysfunction through the NF-κB/HIF-1α pathway by targeting HMOX1 in sepsis.

## Methods

### Ethical statement

All experimental procedures and the use of animals were approved by the Ethics committee on animal experiments of Tongji Hospital Affiliated to Tongji Medical College of Huazhong University of Science and Technology. All efforts were made to minimize the suffering of the included animals.

### Cell culture

CACO-2 cells (Shanghai Suer Biotechnology Co. Ltd., Shanghai, China) were incubated in Dulbecco’s modified eagle medium (DMEM) high sugar medium (Life Technologies Corporation, California, USA) (containing 10% fetal bovine serum, 1% non-essential amino acids (NEAA), 2.5 mg/mL plasmocin), and cultured in a humidified incubator at 37 °C with 5% CO_2_ in air. The medium was replaced every two days. When cell confluency reached 80%, the cells were treated with 0.25% trypsin (Gibco Company, Gaitherburg, MD, USA) and were further subcultured for three generations.

### Dual-luciferase reporter assay

The biology prediction website, *microRNA.org* was used to predict the possible target gene of miR-31 and to obtain the sequence fragments containing the site of action. The DNA content was extracted from human colorectal adenocarcinoma cells (CACO-2 cells) according to the instructions of the PureLink® Genomic DNA Mini Kit (Item No. K182001, Thermo Fisher Scientific, Massachusetts, USA), and the HMOX1 3’UTR wild-type sequence (HMOX1–3’-UTR-wt), and the mutant sequence of HMOX1–3’-UTR (HMOX1–3’-UTR-mut) with a deleted miR-31 binding site were designed. Next, a luciferase reporter plasmid vector was constructed, and the miR-31 mimic was transfected into CACO-2 cells (Shanghai Suer Biotechnology Co., Ltd., Shanghai, China). The sample luciferase activity was detected using a dual-luciferase reporter gene assay reagent. After transfection for 48 h, the medium was removed, and the sample was rinsed twice with phosphate buffer saline (PBS). The cells were treated with passive lysis buffer (PLB) (100 μL/well), shaken slightly for 15 min at room temperature, and then the cell lysate was collected. The program was pre-read for 2 s and the value was read for 10 s. The luciferase assay reagent II (LARII) and Stop & Glo® Reagent (Promega Corporation, Madison, WI, USA) (100 μL/sample intake) were prepared, and added to the luminous tube or plate of the cell lysate (20 μL/sample), and then tested using a bioluminescent detector (Modulus™, Sunnyvale, CA, USA).

### Cell transfection

The 3rd generation of CACO-2 cells were transfected with the miR-31 mimic, the miR-31 inhibitor and the negative control (50 nM of final concentration) in accordance with the instructions of lipofectamine 2000 (Shanghai Heng Fei Biotechnology Co., Ltd., Shanghai, China). After being cultured for 24 ~ 48 h, the expression of HMOX1 gene was detected using reverse transcription quantitative polymerase chain reaction (RT-qPCR) and Western blot analysis. The transfection sequences were synthesized by Shanghai GenePharma Co., Ltd. (Shanghai, China). Primer sequences are shown in Table 1.

**Table 1:** RT-qPCR primer sequences

mRNA	Forward primers (5′-3′)	Reverse primers (5′-3′)
miR-31	CAGCTATGCCAGCATCTTGCCT	ATATGGAACGCTTCACGAATT
NF-κB	GGGCATGGGAATTTCCAACTC	GCAGAAGTAACTTTCCGAGAGG
IκB	TTGCTGAGGCACTTCTGAAAG	TCTGCGTCAAGACTGCTACAC
HIF-1α	GTCGGACAGCCTCACCAAACAG	TAGGTAGTGAGCCACCAGTGTCC
HMOX-1	CTCAAACCTCCAAAAGCC	TCAAAAACCACCCCAACCC
ZO-1	CCATTCTTTGGACCGATTGCTG	TAATGCCCGAGCTCCGATG
Occludin	ACGGTGCCATAGAATGAGATGTTG	CAGCTAGTTGTTCATTTCTGCACCA
GADPH	TTCACCACCATGGAGAAGGC	GGCATGGACTGTGGTCATGA
U6	CTCGCTTCGGCAGCACA	AACGCTTCACGAATTTGCGT

Note: *RT-qPCR* Reverse transcription quantitative polymerase chain reaction, *miR-31* microRNA-31, *NF-κB* nuclear factor-kappa B, *IκB* inhibitor of NF-κB, *HIF-1α* hypoxia inducible factor-1α, *HMOX-1* heme oxygenase 1, *ZO-1* zonula occludens-1, *GADPH* glyceraldehyde-3-phosphate dehydrogenase

### Experiment animals and cecum ligation and perforation (CLP**)** model establishment

Male Sprague-Dawley (SD) rats (weight: 250 ~ 350 g) were provided by the Department of Laboratory Animal Science of Tongji Hospital Affiliated to Tongji Medical College of Huazhong University of Science and Technology. The laboratory animals were fed for more than 3 days for acclimatization, and fasted for 6 h prior to the experiment with free access to water. The CLP model of sepsis was established as follows. A median incision (2 cm) was made in the middle of the abdomen to open the abdominal cavity. The No. 1 silk thread was used for cecal ligation 0.5 cm under the ileocecal valve in the cecum and proximal colon, and then the No.8 syringe needle was perforated through the edge of the mesenterium in the cecum. No. 1 silk thread was left for preventing pinhole from closing. The sham group was underwent dissociation, without ligation and perforation. After concluding the surgery, Ringer’s solution (50 mL/kg) was subcutaneously injected to supplement the loss of intraoperative fluid. Rats in each group were gave free access to food. Positive intestinal microflora was detected in blood about 6 h after the CLP surgery and clinical signs of the sepsis emerged, which suggested modeling success (Rittirsch et al. 2009).

### Animal grouping

The rats were divided into the following 7 groups (*n* = 25): sham group (sham group, 1 ml normal saline was injected intravenously 24 h before the surgery), sepsis group (CLP model rats, 1 ml normal saline was injected intravenously 24 h before the surgery), negative control (NC) group (CLP model rats, 10 μg NC sequence was injected intravenously 24 h before the surgery), miR-31 mimic group (CLP model rats, 10 μg miR-31 mimic was injected intravenously 24 h before the surgery), miR-31 inhibitor group (CLP model rats, 10 μg miR-31 inhibitor was injected intravenously 24 h before the surgery), siRNA-HMOX1 group (CLP model rats, 10 μg siRNA-HOMX1 was injected intravenously 24 h before the surgery), and miR-31 inhibitor + siRNA-HMOX1 group (CLP model rats, 10 μg miR-31 mimic and 10 μg sRNA-HOMX1 were injected intravenously 24 h before the surgery). All siRNA, mimic, and inhibitor were processed by the in vivo RNA reagent (Engreen, 18,668–11-2, Engreen Biosystem Co. Ltd., Beijing, China). The operation was carried out in accordance with the instructions. A total of 15 rats in each group were randomly selected in order to observe the physiological activities and the survival rate at 72 h, and the remaining rats in each group (10 rats for each group) were used for other experiments (Zhao et al. 2016).

### Specimen collection

Twenty-four hours after the surgery, 5 rats were randomly selected from each group, and were anesthetized with 1% pentobarbital injections (0.2 mL/100 g) into the abdominal cavity. The abdominal cavity was incised opened and the abdominal aorta was separated. After separation, blood samples were collected using blood collection needles. The blood was centrifuged at 1812×g for 15 min, and the supernatant was collected to detect serum indexes. Next, the intestinal tissues were incised under aseptic conditions to detect the levels of inflammatory factors and to observe the histological changes. The liver, spleen, mesenteric lymph nodes and ileal tissue of the rats were removed quickly under aseptic condition. Western blot analysis and RT-qPCR were employed in order to test the expression of related genes and proteins.

### Detection of intestinal mucosal permeability function

The serum D-lactic acid levels of rats were tested by coupled liquid chromatography and UV-visible spectrophotometry. The D-lactic acid was oxidized specifically using D-Lactate dehydrogenase, and then the colored oxidation product was produced, which was detected by automatic microplate reader at excitation wavelength of 450 nm. All specific experimental steps were carried out in accordance with the instructions. The absorbance was measured at 450 nm using a microplate reader (Bio-Rad 550, Hercules, CA, USA). A standard curve was plotted, and the levels of D-lactic acid were evaluated. The experiment was conducted three times to obtain the mean values.

The levels of Diamine oxidase (DAO) were tested using enzyme-linked immunosorbent assay (ELISA). All specific experimental steps were conducted according to the instructions. The absorbance was measured at excitation wavelength of 450 nm using a microplate reader (Bio-Rad 550, Hercules, CA, USA). A standard curve was plotted, and the levels of DAO were evaluated. The experiment was conducted three times to obtain the mean value.

Rats from each group were treated with gavage administration of 750 mg/kg FD-40 18 h after the surgery. After gavage administration for 6 h, venous blood samples were collected from the mesentery of rats with the serum separated. The absorbance was measured by a fluorescence spectrophotometer (Beckman, Palo Alto, CA, USA) at excitation wavelength of 490 nm and emission wavelength of 520 nm. A standard curve was plotted, and the levels of FD-40 of the venous blood in mesentery were evaluated. The experiment was conducted three times to obtain the mean value.

### Detection of intestinal barrier function

A total of 5 rats were randomly selected in each group 20 h after modeling, and were treated with gavage administration of 0.6 mg/g FITC-DX (Item No. DX500-BNFC-1, DMD BioMed Ltd., Suzhou, Jiangsu, China) under conditions void of light. Four hours later, the rats were anesthetized and 1 mL blood samples were collected by heart puncture. The obtained blood was centrifuged at 201×g for 10 min at 4 °C, and the supernatant was collected and tested using a fluorescence spectrophotometer (F-7000, Yi De science instrument Co., Ltd., Guangdong, Guangzhou, China) at an excitation wavelength of 490 nm and emission wavelength of 520 nm. A standard curve was plotted, and the levels of FITC-DX were evaluated. The experiment was conducted three times to obtain the mean value.

The liver, spleen, mesenteric lymph nodes of the rats were placed in a sterile homogenizer, and sterile 0.85% sodium chloride solution was added at a 1: 10 ratio to make tissue homogenates. The tissue homogenates (0.1 mL) were collected, and 15 mL blood agar medium at 55 °C were added into a sterile plate, all of which were incubated at 37 °C for 24 h. Then, the organs with bacteria cultured were counted as the positive organs. Next, the rates of bacterial translocation (number of positive organs/total number of cultured organs) were counted. The experiment was conducted three times to obtain the mean value.

### Malondiadehyde (MDA) content and superoxide dismutase (SOD) activity

Intestinal tissues obtained from the sham group or 24 h after CLP were added with 1 mol/L HCI solutions, ground into tissue homogenates, and centrifuged at 1812×g for 10 min to collect the supernatant. In accordance with the instructions of the employed SOD test kit (HL70042, Shanghai haling biotechnology Co., Ltd., Shanghai, China) and MDA test kit (HLTO1013, Shanghai haling biotechnology Co., Ltd., Shanghai, China), the SOD activity and MDA content were evaluated using the colorimetric method by an automatic microplate reader (Beckman Coulter, Fullerton, CA, USA). The cells in each group were collected, and centrifuged after ultrasonic cell disruption. The supernatant (100 μL) was obtained to test the optical density (OD) value using an automatic microplate reader in accordance with the instructions of the SOD and MDA test kit, and the vitalities were calculated. The experiment was conducted three times to obtain the mean value.

### Detection of inflammatory factors

The intestinal tissues obtained from the sham group or 24 h after CLP were treated with a homogenizer. The supernatant was separated after centrifugation at 20128×g for 15 min at 4 °C. The levels of tumor necrosis factor (TNF-α), interleukin (IL)-6, and IL-10 were tested using an ELISA test kit (TWp022566, TWp028583, TWp028605, Shanghai Tong Wei Biological Technology Co., Ltd., Shanghai, China). The operations were carried out in strict accordance with the kit instructions. The test kit was maintained at room temperature for 20 min, and the detergents were prepared. A total of 10 standard wells (including 2 blank control wells, no samples and enzyme labeling reagents) were set on the enzyme-labeled plate, and the standard curve was plotted after standard dilution. The samples were diluted, and then placed into the sample wells of the enzyme-labeled plate. The plates were shaken gently after the addition of samples, and then sealed for incubation for 30 min at 37 °C. The liquid in the wells were removed, detergents were added and removed after 30 s. The process was repeated 5 times, and then the samples were dried. The enzyme labeling reagents (50 μL) was added and incubated for 30 min at 37 °C. Then the liquid in the wells were removed, the detergents were added and removed after 30 s. The process was repeated for 5 times and then the sample was dried. Next, Chromogenic agent A (50 μL) was added to each well, then Chromogenic agent B (50 μL). After gentle mixing, the samples were incubated at 37 °C for 15 min, and 50 μL stop buffer was added. The OD value per well was measured respectively at an excitation wavelength of 450 nm using a microplate reader (Bio-Rad, Hercules, CA, USA) within 10 min with the blank well serving as the control. The concentration standard curve was plotted, and the sample concentration was recorded according to the standard curve. The experiment was conducted three times to obtain the mean value.

### Hematoxylin and eosin (HE) staining

The middle parts of intestinal tissues of rats from each group were fixed with 4% formaldehyde for 6 h, and embedded in paraffin wax. The paraffin-embedded tissues were sliced into 3 μm sections. After being baked at 60 °C, the sections were dewaxed in xylene I and xylene II, for 20 min each time. After that, the sections were placed in 100%, 95%, 80%, 70% ethanol respectively for 5 min and then rinsed with distilled water for 3 min. Next, the samples were stained with hematoxylin for 10 min, washed under tap water for about 15 min, and then stained with eosin for 30 s. The double distilled water was used for washing until red coloration was all washed away. Then the sections were dehydrated in alcohol, cleaned with xylene and sealed with neutral gum. A light microscope was employed for histopathological examination and photographing, and them the tissue coloration and the staining intensity were observed. The pathological damage of rats was scored according to the standard of Chiu’s intestinal injury (Chiu et al. 1970). The experiment was repeated three times to obtain the mean value.

### RT-qPCR

Rats in each group were sacrificed by cervical dislocation, and a section of the ileal tissues was extracted. After analyzing the quality, the RNA content of the sample was extracted using Trizol in accordance with the Trizol instructions (Item: 15596–018, Invitrogen, Carlsbad, CA, USA). The RNA content was dissolved in ultra-pure water treated with diethylpyrocarbonate (DEPC) (A100174–0005, Shanghai Sangon Biotech Co., Ltd., Shanghai, China), and absorbance at 260 nm and 280 nm were measured using an ND-1000 U*V*/Vis spectrophotometer (Thermo Scientific, Massachusetts, CA, USA) in order to evaluate the quality of the total RNA. Reverse transcription of the extracted RNA was completed with a two-step method according to the kit instructions (Thermo Scientific, Massachusetts, CA, USA). The reaction conditions were as follows: 10 min at 70 °C, 2 min in ice bath and then 60 min at 42 °C, and finally 10 min at 70 °C. The cDNA obtained from the reverse transcription was temporarily stored in a refrigerator at − 80 °C. The TaqMan probe method was used in RT-qPCR, and the reaction system was operated according to the kit instructions (MBI Fermentas, Vilnius, Lithuania). The primer sequences are shown in Table 1, and the reaction conditions were as follows: pre-denaturation at 95 °C for 30 s, denaturation at 95 °C for 10 s, annealing at 60 °C for 20 s and extension at 70 °C for 10 s, for a total of 40 cycles. The reaction system was as follows: 12.5 μL Premix Ex Taq or SYBR Green Mix, 1 μL Forward Primer, 1 μL Reverse Primer, 1–4 μL DNA template, and added with ddH_2_O for a total of 25 μL reaction system. A real-time fluorescence quantitative PCR (Bio-Rad, Model Bio-Rad iQ5, Hercules, CA, USA) instrument was for testing. Glyceraldehyde-3-phosphate dehydrogenase (GAPDH) was regarded as the internal reference for the genes. The 2^-ΔΔCt^ method represents the ratio of the expression of the target gene in the experiment group and the control group. The formula was as follows: ΔΔCT = ΔCt _experiment group_ - ΔCt _control group_, ΔCt = Ct _target gene_ - Ct _GADPH_. The experiment was repeated three times to obtain the mean value.

### Western blot analysis

Rats in each group were sacrificed by cervical dislocation, and a section of the ileal tissues were selected and weighed, and then added with 200 μL pre-cooled radio-immunoprecipitation assay (RIPA) lysate (R0020, Beijing Solarbio Life Sciences Co., Ltd., Beijing, China) (containing 1 mmol/L phenylmethylsulfonyl fluoride) The samples were gently shaken to make the cell lysate and the cells in full contact, with the transfer liquid gun treated a few times, and then cracked on ice for 30 min. The protein lysate was placed into a new centrifuge tube, and centrifuged at 289845×g for 5 min at 4 °C with the upper protein extracted. The protein concentration was tested using a bicinchoninic acid (BCA) protein assay kit (AR0146, Boster Biological Technology Co., Ltd., Wuhan, Hubei, China). The protein extracted from the ileal tissue was added to the loading buffer, 30 μg per well, and then boiled at 95 °C for 10 min. The 10% polyacrylamide gel (mc0001, Shanghai McKinang Biotechnology Co., Ltd., Shanghai, China) electrophoresis was used to separate the proteins. After electrophoresis, the protein was transferred to poly (vinylidene fluoride) (PVDF) membrane (P2438, Sigma, St Louis, MO, USA) using the semi-dry electrotransfer method, and the membrane was sealed with 5% bovine serum albumin (BSA) at room temperature for 1 h. Then, the PVDF membrane was incubated with the addition of the following antibodies anti-rabbit monoclonal antibody NF-κB (1: 1000; ab32360), rabbit polyclonal antibody IκB (1: 500; ab64813), rabbit monoclonal antibody HIF-1α (1: 500; ab51608), rabbit polyclonal antibody ZO-1 (1: 100; ab59720), rabbit monoclonal antibody Occludin (1: 50000; ab167161), and the rabbit monoclonal antibody HMOX1 (1: 2000; ab52947) at 4 °C overnight. All the above mentioned antibodies were purchased from Abcam Inc. (Cambridge, MA, USA). The next day, the membrane was washed 3 times with Tris-buffered saline with Tween 20 (TBST) (5 min/time), and the goat anti-rabbit second antibody was added to the membrane for incubation at room temperature for 1 h. And then, the membrane was rinsed 3 times (5 min/time), and developed using a chemiluminescent reagent (Research Biology Co., Ltd., Shanghai, China) with GADPH serving as the internal reference. The Bio-rad Gel Dol EZ Imager (GEL DOC EZ IMAGER, Bio-Rad, Hercules, CA, USA) was employed to analyze the obtained images. The gray scale of the target protein band was analyzed using Image J (National Institutes of Health), and the ratio of the gray value of the target protein band to the gray value of the internal reference protein was used as the levels of the target protein. The experiment was repeated three times to obtain the mean value.

### Statistical analysis

Statistical analyses were processed using SPSS 21.0 software (IBM Corp., Armonk, NY, USA). Measurement data were expressed as mean ± standard deviation. The *t*-test was applied for comparisons between two groups, and one-way analysis of variance (ANOVA) for comparisons among groups. Count data was expressed as a percentage or rate, and the x^2^ test was used for comparison. ANOVA was used for comparisons between multiple groups, and homogeneity test of variances was used. When the ANOVA presented with significant differences, the q test was used for further comparisons, otherwise, the nonparametric rank sum was used, and the significance level α = 0.05. *p* < 0.05 was considered to be statistically significant.

## Results

### miR-31 affects intestinal mucosal permeability and intestinal barrier function

**Fig. 1:**
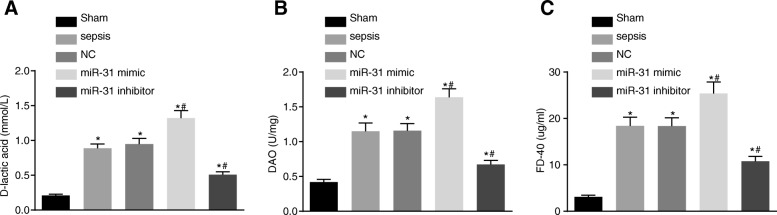
Intestinal mucosa permeability was affected by miR-31 levels. **a** levels of D-lactic acid in serum of rats increased after the treatment of miR-31 mimic; (**b**) levels of DAO in serum of rats elevated after treating with miR-31 mimic; (**c**) levels of FD-40 in serum of rats upregulated by miR-31 mimic; ^*^, *p* < 0.05, vs. the sham group; ^#^, *p* < 0.05, vs. the sepsis group; *NC* negative control, *DAO* Diamine oxidase

Rats were subjected to CPL and administration of during sepsis in order to detect the role of miR-31 in intestinal mucosal permeability. Next, serum samples were obtained 24 h after injection in order to determine the levels of D-lactic acid, DAO and FD-40. The results are shown in Fig. 1. The levels of D-lactic acid, DAO and FD-40 in serum in the other groups were found to be significantly higher than in the sham group (*p* < 0.05). The levels of D-lactic acid, DAO and FD-40 were evidently increased in the miR-31 mimic group (*p* < 0.05), while significantly decreased in the miR-31 inhibitor group in comparison to the sepsis group (*p* < 0.05).

After administration of miR-31 mimic or inhibitor, FITC-DX content and bacterial translocation rate were detected in order to determine the effect of miR-31 on intestinal barrier function (Table 2). The FITC-DX content and the bacterial translocation rate were found to be significantly higher in other groups than those in the sham group (all *p* < 0.05). In addition, the levels of FITC-DX and the bacterial translocation rate were found to be markedly higher in the miR-31 mimic group while obviously lower in the miR-31 inhibitor group when compared with those in the sepsis group. The above results showed that intestinal mucosal permeability and intestinal barrier function could be negatively affected by the level of miR-31.

**Table 2:** Intestinal barrier function of the rats is affected by miR-31

Group	FITC-DX (μg/mL)	Numbers	Visceral organ			Bacterial translocation rate (%)
			Liver	Spleen	Mesenteric lymph node	
Sham	0.27 ± 0.01	5	0	0	1	6.67%
Sepsis	1.93 ± 0.13^*^	5	3	3	4	66.67%^*^
NC	1.99 ± 0.14^*^	5	5	3	3	73.33%^*^
miR-31 mimic	2.71 ± 0.19^*#^	5	4	5	5	93.33%^*^
miR-31 inhibitor	0.78 ± 0.06^*#^	5	5	2	2	40.00^*^

Notes: ^*^, *p* < 0.05, vs. the sham group; ^#^, *p* < 0.05, vs. the sepsis group; *NC* negative control

### Levels of miR-31 can cause oxidative damage and affect the expression of inflammatory factors in intestinal tissue of rats

**Fig. 2:**
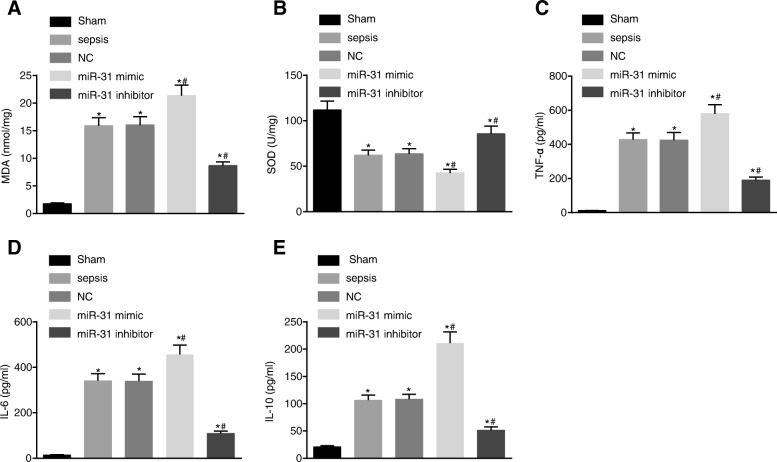
MiR-31 caused oxidative damage and affected the levels of inflammatory factors in intestinal tissue of rats. **a** content of MDA increased after treating with miR-31 mimic; (**b**) SOD activity was inhibited by miR-31 mimic; (**c**) elevated levels of TNF-α after the treatment of miR-31 mimic; (**d**) increased levels of IL-6 following the treatment of miR-31 mimic; (**e**), increased levels of IL-10 by miR-31 mimic; ^*^, *p* < 0.05, vs. the sham group; ^#^, *p* < 0.05, vs. the sepsis group; *MDA* malondiadehyde, *SOD* superoxidedismutase, *NC* negative control, *TNF* tumor necrosis factor, *IL* interleukin

Additionally, the MDA content and SOD activity were measured in order to detect the effect of miR-31 on oxidative damage of intestinal tissue of rats. To further study the oxidative damage caused by miR-31 in rat small intestine tissue, MDA was regarded as an indicator to indirectly reflect the damage of oxygen free radicals to cells, and SOD to reflect the antioxidant capacity of the cells. The results showed that the MDA content in the intestine tissues in other groups was found to be significantly higher while the SOD activity significantly was lower than that in the sham group (all *p* < 0.05). Compared with the sepsis group, the MDA content in the intestine tissues was evidently increased and the SOD activity was significantly decreased in the miR-31 mimic group (all *p* < 0.05), while the miR-31 inhibitor group exhibited opposite trends (all *p* < 0.05) (Fig. 2a and b).

Furthermore, the contents of TNF-α, IL-6 and IL-10 were detected in order to study the inflammation induced by miR-31 on the small intestine of rats. The levels of TNF-α, IL-6 and IL-10 in the intestine tissues in each group are shown in Fig. 2c-e. The levels of TNF-α and IL-6 and IL-10 in the intestine tissues in the other groups were found to be significantly higher than those in the sham group (all *p* < 0.05). The levels of TNF-α, IL-6 and IL-10 were significantly higher in the miR-31 mimic group (all *p* < 0.05) while significantly lower levels were observed in the miR-31 inhibitor group when compared with the sepsis group (all *p* < 0.05). The above results showed that oxidative damage of intestinal tissue of rats could be caused by treatment with miR-31, and the expression of inflammatory factors in intestinal tissue of rats was also regulated by miR-31.

### Confirmation of HMOX1 as a target gene of miR-31

**Fig. 3:**
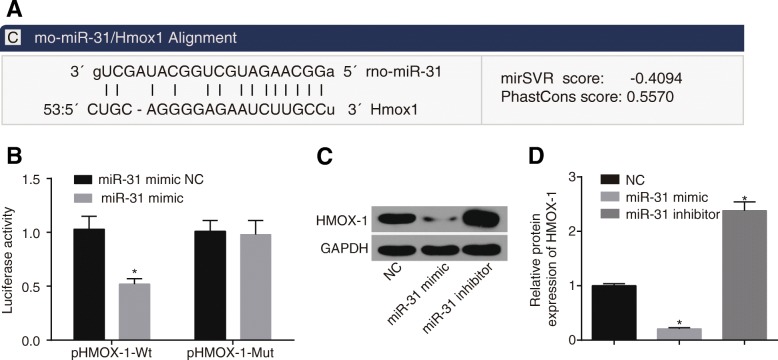
HMOX1 was the target gene of miR-31. **a** HMOX1 was confirmed as a target gene of miR-31 by the biology predicted website microRNA.org; (**b**) dual-luciferase reporter assay showed HMOX1 was a target gene of miR-31; (**c**) Western blot analysis detected that protein levels of HMOX1 were significantly increased after treating miR-31 inhibitor; (**d**) inhibition of miR-31 could increase protein levels of HMOX1; ^*^, *p* < 0.05, vs. the corresponding control group

HMOX1 was confirmed as a target gene of miR-31 by the biology predicted website, *microRNA.org* (Fig. 3a). The results of dual-luciferase reporter gene assay (Fig. 3b) showed that the fluorescence signal of the miR-31 mimic + pHMOX1-Wt group was decreased by approximately 50% (all *p* < 0.05) when compared with the other three groups, and the miR-31 mimic + pHMOX1-Mut presented with no significant differences in the luciferase signal compared with the miR-31 mimic NC + pHMOX1-Mut group and the miR-31 mimic NC + pHMOX1-Wt group (all *p* > 0.05). In addition, western blot analysis was applied in order to detect the expression of HMOX1 in CACO-2 cells. It was found that (Fig. 3c, d) compared with the NC group, the expression of HMOX1 in the miR-31 mimic group was significantly down-regulated (*p* < 0.05), while obviously up-regulated in the miR-31 inhibitor group (*p* < 0.05). These findings suggested that miR-31 could target HMOX1.

### MiR-31 affects intestinal mucosal permeability and intestinal barrier function by regulating HMOX1

**Fig. 4:**
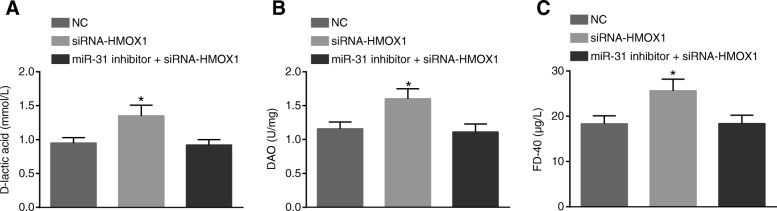
MiR-31 can influence intestinal mucosa permeability through regulation of HMOX1. **a** increased levels of D-lactic acid in serum of rats after the treatment of siRNA-HMOX1; (**b**) siRNA-HMOX1 could increase levels of DAO in serum of rats; (**c**) increased levels of FD-40 in serum of rats by siRNA-HMOX1; ^*^, *p* < 0.05, vs. the NC group; *NC* negative control, *DAO* Diamine oxidase

In order to further study the effect of miR-31 on intestinal mucosal permeability and intestinal barrier function in rats by regulating HMOX1, we interfered with HMOX1 with siRNA. The detection results of intestinal mucosal permeability function are shown in Fig. 4. Compared with the NC group, the contents of D-lactic acid, DAO and FD-40 in serum were found to be significantly increased in the siRNA-HMOX1 group (all *p* < 0.05). There were no significant differences in the contents of D-lactic acid, DAO and FD-40 between the miR-31 inhibitor + siRNA-HMOX1 group and the NC group (*p* > 0.05).

The results of intestinal barrier function in each group are shown in Table 3. Compared with the NC group, the siRNA-HMOX1 group presented with elevated FITC-DX content and increased bacterial translocation rate in each organ. There were no obvious differences in FITC-DX content and bacterial translocation rate between the miR-31 inhibitor + siRNA-HMOX1 group and the NC group (all *p* > 0.05). These results suggested that intestinal mucosal permeability and intestinal barrier function could be influenced by miR-31 through the regulation of HMOX1.

**Table 3:** MiR-31 influences intestinal barrier function of the rats by regulating HMOX1

Group	FITC-DX (μg/mL)	Numbers	Visceral organ			Bacterial translocation rate (%)
			Liver	Spleen	Mesenteric lymph node	
NC	1.99 ± 0.14	5	5	3	3	73.33%
siRNA-HMOX1	2.83 ± 0.21*	5	5	5	5	100%*
miR-31 inhibitor + siRNA-HMOX1	1.96 ± 0.11	5	5	2	3	66.67%

Note: ^*^, *p* < 0.05, vs. the NC group; *NC* negative control, *HMOX1* heme oxygenase 1

### MiR-31 affects oxidative damage and inflammation level in rat small intestine tissues through HMOX1

**Fig. 5:**
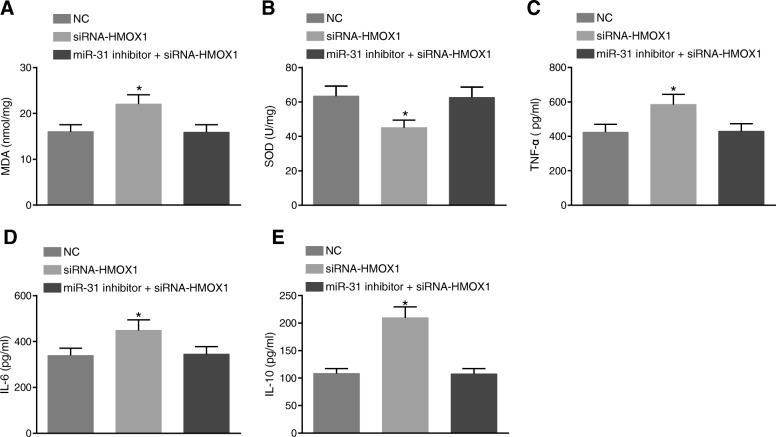
MiR-31 affects oxidative damage and the levels of inflammatory factors in intestinal tissue of rats by regulating HMOX1. **a** siRNA-HMOX1 increased content of MDA; (**b**), SOD activity was down-regulated by siRNA-HMOX1; (**c**) increased levels of TNF-α after the treatment of siRNA-HMOX1; (**d**) increased levels of IL-6 following the treatment of siRNA-HMOX1; (**e**) increased levels of IL-10 by siRNA-HMOX1; ^*^, *p* < 0.05, vs. the NC group; MDA, malondiadehyde; SOD, superoxidedismutase; *NC* negative control, *TNF* tumor necrosis factor, *IL* interleukin

The content of MDA and SOD activity in the small intestine of rats was measured in order to detect oxidative damage. The results are shown in Fig. 5a and b. In comparison to the NC group, the content of MDA was found to be remarkably increased while SOD activity was obviously reduced in the siRNA-HMOX1 group (all *p* < 0.05). There were no significant differences in the content of MDA and SOD activity between the miR-31 inhibitor + siRNA-HMOX1 group and the NC group (*p* > 0.05). These findings showed that miR-31 can affect MDA content and SOD activity in rat small intestine through HMOX-1.

In addition, the content of TNF-α, IL-6 and IL-10 in rats was detected in order to study the inflammation induced by miR-31 on the small intestine of rats through HMOX1. The results are shown in Fig. 5c-e. In comparison to the NC group, the siRNA-HMOX1 group presented with significantly increased TNF-α, IL-6 and IL-10 content in rats (all *p* < 0.05). There were no significant differences in the contents of TNF-α, IL-6 and IL-10 between the miR-31 inhibitor + siRNA-HMOX1 group and the NC group (*p* > 0.05). These findings showed that miR-31 can induce oxidative damage in rat small intestine, and furthermore affect the expression of inflammatory factors through HMOX-1.

### Relatively mild histopathological changes in intestine tissues after transfection with miR-31 inhibitor

**Fig. 6:**
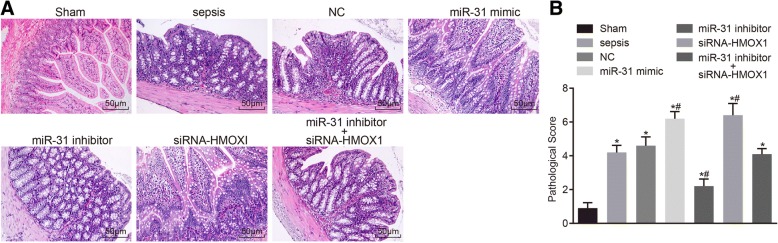
MiR-31 resulted in histopathological changes in intestine tissues of rats. **a** histopathological changes of the intestine tissues of the rats could be caused by miR-31 (HE staining 200 ×); (**b**) pathological injury of small intestine tissue in rats was abated by inhibiting miR-31; ^*^, *p* < 0.05, vs. the sham group; ^#^, *p* < 0.05, vs. the sepsis group; *NC* negative control, *HE* hematoxylin and eosin

HE staining was employed in order to detect histopathological changes of the intestine tissues in rats. The results of HE staining in the intestine tissues in each group are shown in Fig. 6. The rats in the sham group exhibited normally structured intestines under light microscope observation, without tissue edema, and presented with normal villus structure and clear edge of microvilli, with a pathological injury score of 0.90 ± 0.32. In the sepsis group, the intestinal wall was noted to be thinner, the mucosa was atrophied, in addition to intestinal mucosal necrosis, shedding and occurrence of villous rupture in some areas, as well as a pathological injury score of 4.20 ± 0.42, which was significantly different compared with the sham group (*p* < 0.05). The histopathological changes of the rats in the NC group and the miR-31 inhibitor + siRNA-HMOX1 group were consistent with the sepsis group, with pathological injury scores of 4.60 ± 0.52 and 4.10 ± 0.32, respectively. The intestinal wall and mucosal atrophy of rats in the miR-31 mimic group and the siRNA-HMOX1 group were more obvious, with more intestinal mucosal necrosis, shedding and occurrence of villous rupture in more areas, the pathological injury scores of 6.20 ± 0.42 and 6.40 ± 0.70, respectively. There were statistically significant differences compared with the sham and sepsis groups (*p* < 0.05). The rats in the miR-31 inhibitor group exhibited partial intestinal mucosal necrosis and shedding, but the intestinal mucosal lesions were relatively mild, with a pathological injury score of 2.20 ± 0.42. The above results suggested that miR-31 could cause the above mentioned histopathological changes of the intestine tissues.

### Inhibition of HMOX1 expression by miR-31 affects the expression of NF-κB/HIF-1α pathway related genes

**Fig. 7:**
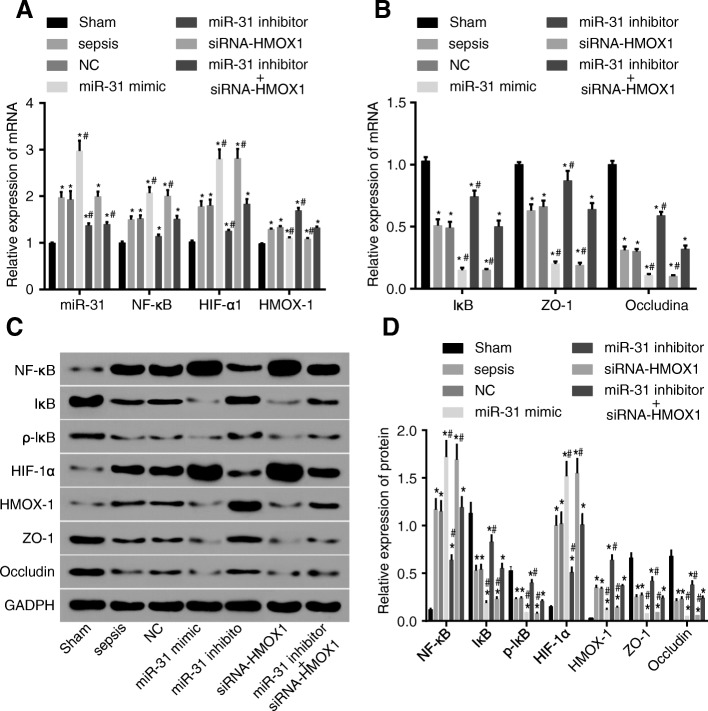
MiR-31 regulated expression of the NF-κB/HIF-1α pathway related genes by inhibiting HMOX1. **a**-**b** RT-qPCR detected that the mRNA levels of NF-κB, HIF-1α, HMOX1, IκB, ZO-1, and Occludin in the ileal tissue of the rats were affected by miR-31 through HMOX1; (**c**-**d**), Western blot analysis detected that protein levels of IκB, p-IκB, ZO-1, HMOX1, HIF-1α, NF-κB, and Occludin in the ileal tissue of the rats were regulated by miR-31 via HMOX1; ^*^, *p* < 0.05, vs. the sham group; ^#^, *p* < 0.05, vs. the sepsis group; *NC* negative control

RT-qPCR and western blot analysis were employed in order to determine the mRNA and protein levels of the NF-κB/HIF-1α pathway related. The levels of miR-31 and mRNA levels of NF-κB, HIF-1α, and HMOX1 in ileal tissues of each group are shown in Fig. 7. The levels of miR-31 in the ileal tissues in the other groups were found to be significantly increased compared to that in the sham group (all *p* < 0.05). The levels of miR-31 were significantly higher in the miR-31 mimic group than in the sepsis group, and significantly decreased in the miR-31 inhibitor and miR-31 inhibitor + siRNA-HMOX1 groups (all *p* < 0.05). Compared with the sham group, the expression of NF-κB and HIF-1α mRNA and protein in ileal tissues were significantly higher in the other groups (all *p* < 0.05), in addition to significantly decreased mRNA and protein levels of IκB, ZO-1 and Occludin (all *p* < 0.05). Compared with the sepsis group, the expression of NF-κB and HIF-1α mRNA and protein in ileal tissues were found to be significantly increased, while IκB, ZO-1 and Occludin mRNA and protein were significantly decreased in the miR-31 mimic group and the siRNA-HMOX1 group (all *p* < 0.05). The expression of NF-κB and HIF-1α mRNA and protein in ileal tissues were found to be remarkably decreased, and IκB, ZO-1 and Occludin mRNA and protein were significantly increased in the miR-31 inhibitor group (all *p* < 0.05). Compared with the sham group, the mRNA and protein levels of HMOX1 in the ileal tissues in other groups were evidently elevated (all *p* < 0.05). In comparison to the sepsis group, the expression of HMOX1 mRNA and protein in the ileal tissues in the miR-31 mimic and siRNA-HMOX1 groups were significantly lower (all *p* < 0.05), whereas significantly higher levels were observed in the miR-31 inhibitor group. The mRNA and protein levels of relative genes in the ileal tissues in the sepsis, NC, and miR-31 inhibitor + siRNA-HMOX1 groups exhibited no significant differences (all *p* > 0.05). The mRNA and protein levels of NF-κB, HIF-1α, HMOX1, IκB, ZO-1, and Occludin (except miR-31) in the ileal tissues in the miR-31 mimic group and siRNA-HMOX1 group presented with no significant differences (all *p* > 0.05). The above results showed that the levels of the NF-κB/HIF-1α pathway related genes could be affected by miR-31 through inhibition of HMOX1.

### Inhibition of miR-31 can increase survival rates of rats

**Fig. 8:**
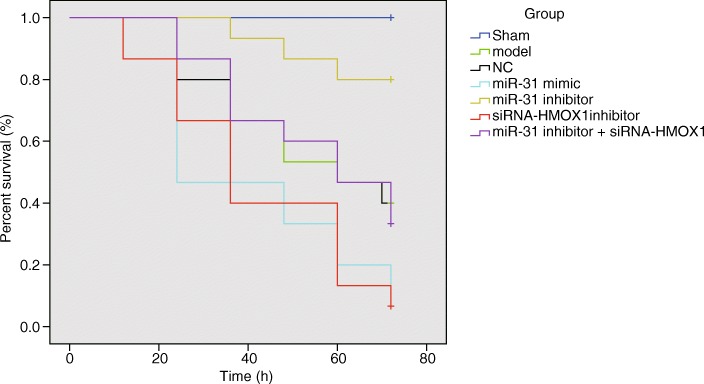
MiR-31 reduced the survival rates of rats. *n* = 15

The rats in the sham group were awoken after anesthetization and fed with a normal diet, and the rats presented with normal stool and body temperature, smooth and shiny hair, and quick response. When the sepsis rats were awoken after anesthetization, the rats were observed to be dull, slow, and presenting with shortness of breath and bloating. Then, the rats died and accompanied by different symptoms during the process. The survival curves of rats at 0–72 h in each group are shown in Fig. 8. The non-parametric estimation of survival rates was based on the Kaplan-Meier method. The median survival time and the 95% confidence interval (CI) of each group were as follows: the sham group, no death; the sepsis group, 95% CI (44.458–64.342); the NC group, 95% CI (43.909–64.624); the miR-31 mimic group, 95% CI (28.571–51.429); the miR-31 inhibitor group, 95% CI (61.860–72.540); the siRNA-HMOX group, 95% CI (31.088–52.112); the miR-31 inhibitor + siRNA-HMOX1 group, 95% CI (45.338–65.062).

The survival curves of the different groups were compared using the Log-rank test. The survival analysis showed that the survival curves in the seven groups were different. There were no deaths in the sham group. Compared with the sham group, the survival rates of rats in other groups were found to be reduced significantly. There were rat deaths at the 12 h time interval in the miR-31 mimic and siRNA-HMOX1 groups, and the number of deaths increased and survival rate decreased with time. In the miR-31 inhibitor group, rat deaths were observed at the 36 h time interval. In the sepsis, NC and miR-31 inhibitor + siRNA-HMOX1 groups, the rats died at the 24 h time interval. These findings suggested that inhibition of miR-31 reduced survival rates of rats.

## Discussion

Sepsis, a life-threatening condition, is arisen when the body’s response to infection results in injury to its own tissues and organs, causing multiple organ dysfunctions like intestinal barrier dysfunction (Ma et al. 2013; Singer et al. 2016). Previously, miRNAs have been regarded to be essential for the regulation of numerous cellular processes, including inflammation and immunity (Ma et al. 2013). The current study aimed to investigate whether miR-31 has effects on intestinal barrier dysfunction by targeting HMOX1 through the NF-κB/HIF-1α pathway in sepsis. The results of our study demonstrated that intestinal barrier dysfunction can be improved through suppressing the expression of miR-31 by targeting HMOX1 through inhibition of the NF-κB/HIF-1α pathway.

Firstly, the current study found that sepsis rats exhibited increased expressions of D-lactic acid, DAO, bacterial translocation, MDA, TNF-α, IL-6, and IL-10, in addition to decreased SOD activity. D-lactic acid is a metabolic product of intestinal flora, and a recent study has shown that patients suffering from chronic fatigue syndrome presented with increased D-lactic acid intestinal bacteria (Sheedy et al. 2009). Serum DAO, acting as a marker for mammalian intestinal mucosa, was found to be significantly higher in rats with irritable bowel syndrome as noted by increased DAO activity (Liu et al. 2012). The intestines are a primary source for endogenous bacteria, and recent evidences have shown that elevated bacterial translocation rates are found in intestinal barrier dysfunction (Gomez-Hurtado et al. 2011). Similarly, decreased SOD activity with increased MDA levels were observed for intestinal barrier function damage in pancreatitis rats (Mirmalek et al. 2016). Interestingly, a previous study suggested that intestinal epithelial barrier dysfunction is triggered by a mechanism that involves mast cell dependent TNF-α and protease release and disruption of paracellular tight junctions, owing to findings of increased TNF-α in intestinal epithelial barrier function (Overman et al. 2012). In addition, data indicated that for obvious pathological damages in the intestine, the levels of TNF-α, IL-6, and IL-10 were elevated (Zhongkai et al. 2012).

Additionally, the current study showed that the miR-31 inhibitor group exhibited decreased expressions of D-lactic acid, DAO, FITC-DX, TNF-α, IL-6, and IL-10, in addition to increased expressions of SOD. A recent study showed that damage to the intestinal mucosa barrier may result in intestinal bacterial and endotoxin translocation, leading to local and systemic inflammation, and furthermore, FITC-DX and D-lactic acid were respectively the markers for changes in intestinal permeability and intestinal barriers (Bao et al. 2014). Similarly, the expression of inflammatory cytokines like TNF-α, DAO and D-lactic acid, and IL-1 were found to be significantly lower in normal conditions in intestinal mucosa compared to intestinal mucosal injury (Shu et al. 2016) and inflammatory cytokines also included IL-6, IL-8, IL-10, and TNF-α (Piantadosi et al. 2011; Terasaka et al. 2010). It was also reported that inhibition of endogenous miR-31 in psoriasis (a common chronic inflammatory skin disease) inhibits the production of inflammatory mediators (Xu et al. 2013). Thus, in the current study, it was assumed that miR-31 was related to intestinal mucosal permeability function and intestinal barrier function. Moreover, another study found that HO-1 and CORM-2 exerted a protective role in intestinal epithelial barrier function by reducing cell apoptosis and intestinal inflammation through regulation of NF-κB (Zhang et al. 2017). Furthermore, increasing evidences have shown that the activity of DAO was high in intestinal villous cells, and reduced activity was noted if injury to intestinal epithelial cells had ensued, so changes of intestinal mucosa integrity, permeability and the barrier function can be shown by DAO activity (Han et al. 2016). Another study also indicated that the expression of SOD in the cytosol was down-regulated by miR-398 during growth on low copper (Higashi et al. 2013).

Furthermore, the current study showed that the rats with sepsis exhibited higher expressions of miR-31, HMOX1, NF-κB, and HIF-1α in addition to decreased expressions of IκB, ZO-1, and Occludin. Whereas, the rats transfected with miR-31 inhibitor presented with higher expressions of IκB, ZO-1, Occludin, and HMOX1 and lower expressions of miR-31, NF-κB, and HIF-1α. Previously, it has been found that miR-31 is expressed widely in different cell types, and has been studied in different diseases (Peng et al. 2012). It was also demonstrated that miR-31 expression is up-regulated in human cervical, colorectal, liver, and head-and-neck squamous cell carcinomas, indicating the role of miR-31 in various cancers (Guo et al. 2016). Additionally, miR-31 was found to be up-regulated in colorectal cancer (Slaby et al. 2007). Indicating correlations, miR-31 was found to negatively regulate the noncanonical NF-κB pathway, and higher expression of Polycomb proteins resulted in lower expression of miR-31 in an epigenetic fashion, causing activation of NF-κB and apoptosis resistance (Yamagishi et al. 2012). miR-31 contributes to colorectal cancer development by targeting factor inhibiting HIF-1α (Chen et al. 2014). In addition, the expression of IκB was found to be decreased in cardiac dysfunction in mice with chronic kidney disease, and lung inflammation and systemic inflammatory response resulted from lipopolysaccharide administration (Chen et al. 2017). As previously reported, it was found an impaired tight junction and decreased expression of occludin and ZO-1 in the intestinal epithelial cells (Yu et al. 2016). Moreover, inhibition of miR-122a increased intestinal occludin expression and protected mice from alcoholic liver disease (Zhao et al. 2015). From the previous studies, induction of HMOX1 by inflammation like in sepsis, is related both to an anti-inflammatory response and to mitochondrial biogenesis (Piantadosi et al. 2011), and HMOX1 can promote pro-inflammatory cytokine secretion and suppress inflammation-induced phenotypic maturation in the immune effector cells and enhance anti-inflammatory cytokine production (Ozen et al. 2015). Another study also reported that over-expression of HO-1 promoted sepsis induced immunosuppression at the late stages of sepsis by promoting polarization of anti-inflammatory gene Th2 and Treg function to inhibit excessive immune responses and maintain tolerance to self-antigens (Yoon et al. 2017). HMOX1 was confirmed as a target gene of miR-31 by dual-luciferase reporter assay. Therefore, the findings of the current study showed that suppression of miR-31 alleviates intestinal barrier dysfunction by targeting HMOX1.

## Conclusion

In conclusion, the current study demonstrated that inhibition of miR-31 exerted positive effects on intestinal barrier dysfunction in sepsis, and HMOX1 was the target gene of miR-31. It should be noted that the specific mechanism of targeted correlation required further exploration. However, it can be expected to be a new target gene for treating intestinal barrier dysfunction in sepsis in order to raise the quality of life of patients suffering from this disease.
